# The chronic care for age-related macular degeneration study (CHARMED): Study protocol for a randomized controlled trial

**DOI:** 10.1186/1745-6215-12-221

**Published:** 2011-10-11

**Authors:** Anja Frei, Katja Woitzek, Mathyas Wang, Ulrike Held, Thomas Rosemann

**Affiliations:** 1 Institute of General Practice and Health Services Research, University of Zurich, Pestalozzistrasse 24, 8091 Zurich, Switzerland; 2 Horten Centre for Patient-oriented Research, University of Zurich, Pestalozzistrasse 24, 8091 Zurich, Switzerland

## Abstract

**Background:**

Neovascular age-related macular degeneration is the leading cause of irreversible blindness in people 50 years of age or older in the developed world. As in other chronic diseases, several effective treatments are available, but in clinical daily practice there is an evidence performance gap. The Chronic Care Model represents an evidence-based framework for the care of chronically ill patients and aims at closing that gap. However, no data are available regarding patients with neovascular age-related macular degeneration.

**Methods/Design:**

CHARMED is a multicenter randomized controlled trial. The study challenges the hypothesis that the implementation of core elements of the Chronic Care Model (patient empowerment, delivering evidence based information, clinical information system, reminder system with structured follow up and frequent monitoring) via a specially trained Chronic Care Coach in Swiss centres for neovascular age-related macular degeneration results in better visual acuity (primary outcome) and an increased disease specific quality of life (secondary outcome) in patients with neovascular age-related macular degeneration. According to the power calculation, a total sample size of 352 patients is needed (drop out rate of 25%). 14 specialised medical doctors from leading ophtalmologic centres in Switzerland will include 25 patients. In each centre, a Chronic Care Coach will provide disease specific care according to the Chronic Care Model for intervention group. Patients from the control group will be treated as usual. Baseline measurements will be taken in month III - XII, starting in March 2011. Follow-up data will be collected after 6 months and 1 year.

**Discussion:**

Multiple studies have shown that implementing Chronic Care Model elements improve clinical outcomes as well as process parameters in different chronic diseases as osteoarthritis, depression or e.g. the cardiovascular risk profile of diabetes patients. This study will be the first to assess this approach in neovascular age-related macular degeneration. If our hypothesis will be confirmed, the implementation of this approach in routine care for patients with with neovascular age-related macular degeneration should be considered.

**Trial Registration:**

Current controlled trials ISRCTN32507927.

## Background

According to estimates of the World Health Organization, chronic diseases will represent the major challenge for health care systems in the developed world [1]. Due to demographic changes, the prevalence of chronic diseases will rise up to a prevalence of 60%. Faced with this tremendous development, evidence based approaches for the care for chronically ills are the purpose of many research projects. Based on the aggregated evidence, Wagner and colleagues developed the Chronic Care Model (CCM) with 6 key dimensions of care (organization of health care, clinical information systems, delivery system design, decision support, self-management support, community resources) as a conceptual framework for the care for chronically ills [2-5]. The aim of the Chronic Care Coach (CCC) is to integrate all evidence-based concepts or approaches into this conceptual framework. One of the major problems addressed by the CCM is the fact that current care of chronically ills is often reactive and triggered by actual problems instead of being proactive, structured and planned [6]. Core elements of the CCM are patient empowerment, providing evidence based information for patients and physicians, as well as frequent monitoring and structured follow up. Several studies have shown that nurse practitioners or specially trained practice can carry out most of these tasks. Especially telephone based follow up and case management has been proven to be an effective and efficient approach to ensure appropriate monitoring, especially regarding chronic diseases. Several studies reported significant improvements in process parameters as well as in clinical outcomes in common diseases as e.g. diabetes, osteoarthritis and even depression care [7-9].

Neovascular age-related macular degeneration (wet AMD) is a typical chronic disease and the leading cause of irreversible blindness in people 50 years of age or older in the developed world [10,11]. As in other chronic diseases, several effective treatments are available, but clinical experience has shown that these treatments are not as frequently provided as clinical evidence suggests [12]. Daily practice is characterized by an evidence performance gap. Wet AMD as well as other mostly age-related causes of ocular morbidity are on the rise in all industrialized countries and associated with a tremendous loss of quality of life [13,14]. Since wet AMD is a chronic disease, it can be hypothesized that treatment approaches focussing on a structured monitoring and follow-up may also result in improved outcomes in wet AMD.

This study challenges the hypothesis that the implementation of core elements of the CCM (patient empowerment, delivering evidence based information, clinical information system, reminder system with structured follow up and frequent monitoring) results in better visual acuity in patients suffering from wet AMD, an increased disease specific quality of life (outcomes), mediated by a better treatment adherence.

## Methods

### Trial design

**Figure 1:**
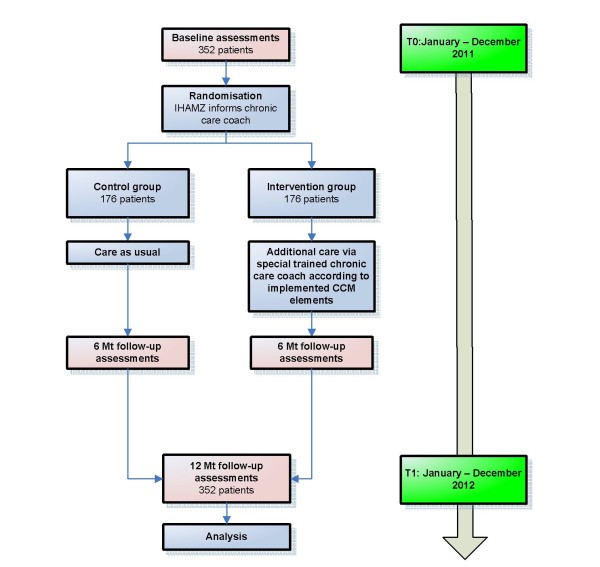
**Flow chart**.

A multicenter randomized controlled trial with the patient as the unit of randomization. The flow chart of the study is described in Figure 1.

### Participants

All eligible patients with wet AMD who are treated in selected centres in Switzerland will be addressed consecutively by the physicians and informed about the study with written material.

### Inclusion criteria for patients

■ Male or female patients with wet AMD

■ Eligible for a therapy with anti-angiogenic drug

■ Visual acuity ≥ 0.05 (assessed by Early Treatment Diabetic Retinopathy Study [ETDRS] charts)

■ Age > 50 years

Written informed consent given before any study related procedure is performed

### Exclusion criteria for patients

■ Serious general or psychological illness (advanced malignant tumours, serious depressive episodes, evidence of dementia)

■ Insufficient language skills (informed consent, patient information and questionnaires will be provided in German and French)

■ Patients with any invasive medical treatment for wet AMD in the past

### Intervention

The aim of the intervention is to implement established, evidence based core elements of the CCM in the care for patients with wet AMD. Wet AMD is as diabetes, asthma, hypertension etc. a chronic disease with evidence (according to guidelines) that frequent monitoring/follow ups and treatments as needed increase visual acuity and quality of life. In daily practice frequent follow ups and monitoring are often lacking. Therefore, in each centre a specially trained chronic care coach (in half-day workshops) will monitor the treatment, including telephone reminders, patient information and direct self-management support in the centre. Evidence based information on the treatment and the need for a monthly follow up will be provided to patients. Patient empowerment according to established programs will be provided during meetings with professionals, peer group meetings and by peer contact persons.

In detail the key elements of the CCM will be addressed as follows:

#### Organization of health care delivery system

The specially trained CCCs will monitor and organize the treatment and structure and plan the contacts between patients and physicians. They will call the patient monthly, if necessary more frequently upon request of the patient. During the calls, the self-measured data of the visual acuity with the Amsler-test and the Health Management Tool (HMT), assessed by iPhone, will be asked. The HMT measures retinal visual function via a contrast contour test. To perform these phone calls and assessments, the CCCs will be trained in one day sessions. Since the CCCs are also involved in the treatment of patients in the control group, a "leakage" of information about the CCM cannot completely be avoided. But the main elements of the intervention (iPhone, phone calls from the CCC, peer group meeting) are definitely only accessible to patients within the study group.

#### Self-management support

Self management is an important issue in chronic diseases. Several interventions will be provided to patients in this study to increase their self management:

1. Initially, patients will be individually taught by the CCC. The CCC will also be the contact person at the study centre in case of further queries. Patients will be instructed to measure the visual acuity of both eyes weekly (with the Amsler-test and the HMT). All patients will be instructed initially by the CCC on how to handle these tools. To assess the efficacy of the HMT, all contacts initiated from the patient due to the self assessment of visual acuity by means of the HMT will be assessed separately.

2. Patients will receive an action plan, which will tell them how to deal with the disease, to estimate the severity of symptoms for subsequent needed actions and how to react if they recognize any changes in the visual acuity. Furthermore, the action plan contains an overview of the planned visits and contact information of the CCC.

3. Peer group meetings are known to be effective interventions; therefore, peer group meetings with experienced patients suffering from wet AMD will take place at least twice in collaboration with Retina Suisse, the Swiss patient organisation for retinal diseases. Peer contacts (patients with wet AMD who are available for queries) will be able to support patients additionally.

#### Decision support

Evidence-based information, based on published guidelines and patient information leaflets will be provided with detailed information about what to do ("Don't worry" leaflet and "Call immediately" leaflets), including a checklist for the antibiotic eye drops and all important contact addresses. Also a timetable to conduct the HMT and the Amsler self-tests will be provided to the patients.

#### Delivery system design and Clinical information systems

Appropriate IT and data bases ease a frequent and structured follow-up. The positive effect of reminder systems on the quality of care for chronically ills has been well documented. Therefore, a computer based reminder system with structured follow up and frequent monitoring will be implemented in all centres to organise the monthly visits for injections and the monthly scheduled phone calls but used only for the monitoring of the patients in the intervention group. The first 3 dates will be fixed with this system automatically.

### Outcomes

The outcome parameters and instruments which are used in the study are summarized in tables 1, 2 and 3.

**Table 1:** Assessment by physicians/clinical visits in the centres = > assessed by questionnaire

	Baseline	After 6 months^1)^	After 12 months
Visual acuity: ETDRS	x	x	x

Central retinal thickness: OCT	x	x	x

Comorbidities	x		x

Family anamnesis of wet AMD	x		x

Smoking	x		x

Medication	x		x

ACIC^2)^	x		x

Outcome-parameters and time of assessment of the study

1) Additional questionnaire 6 month only with ETDRS and OCT assessment

2) Additional questionnaire

**Table 2:** Assessment by chronic care coach by telephone monitoring

	Baseline	M 2	M 3	M 4	M 5	**After 6 month**s	M 7	M 8	M 9	M 10	M 11	After 12 months
NEI VFQ-25^1)^	x					x						x

Self-measured visual acuity test: Amsler-test (weekly, at home)	x	x	x	x	x	x	x	x	x	x	x	x

Retinal visual function assessments by iPhone (contrast contour test, weekly, at home)	x	x	x	x	x	x	x	x	x	x	x	x

Outcome-parameters and time of assessment of the study

1) Interviewer version (assessed if possible in direct patient contact, if not by telephone)

**Table 3:** Assessments by patient questionnaire

	Baseline	After 6 months	After 12 months
Health services utilisation	x		x

PHQ-9	x		x

PACIC	x		x

Sociodemographics	x		x

Outcome-parameters and time of assessment of the study

Abbreviations for the tables:

EDTRS: Early Treatment Diabetic Retinopathy Study

OCT: Optical Coherence Tomography

Wet AMD: Neovascular age-related macular degeneration

ACIC: Assessment of Chronic Illness Care

NEI VFQ-25: National Eye Institute Visual Function Questionnaire-25

PHQ-9: Prime MD Patient Health Questionnaire (Depression Scale)

PACIC: Patient Assessment of Chronic Illness Care

### Primary outcome

The primary outcome is the visual acuity. The measurements will be taken under standardised conditions in the centres in a sitting position at an initial test distance of 4 meters using ETDRS charts [15,16]. To assure standardized measurements, each study centre will be visited and teached accordingly by our study team. Each study centre will be controlled on outreach visits at minimum once within the study period. Besides the name of the equipment used in each study centre will be listed in our study data.

The chart has five letters of the same size per row and 14 rows it total, and the letters of the following rows become gradually smaller (with a difference of 0.1 logMAR). The outcome value will be the number of letters correctly read by the patient. The power calculation is based on the ETDRS.

### Secondary outcomes

#### Disease specific quality of life

Disease specific quality of life will be assessed by a well established patient-reported outcome measure, the National Eye Institute Visual Function Questionnare-25, interviewer version (NEI VFQ-25) [17,18]. The NEI VFQ-25 was developed based on qualitative research with patients to measure the range of vision-related functioning. It contains 12 subscales: general vision, near vision, distance vision, driving, peripheral vision, colour vision, ocular pain, general health, vision specific role difficulties, dependency, social function, and metal health. Subscale scores are calculated by summing the appropriate items and transforming the raw scores into a 0 to 100 scale (higher scores indicate better functioning or well-being), the total score is an average of 11 subscale scores, excluding the single item general health subscale. The NEI VFQ-25 has shown to be a valid and easy to use instrument, showing a high correlation with the visual acuity [19-21]. The German and French translation of the NEI VFQ-25 has been validated [22,23].

#### Physiological outcome

The central retinal thickness will be routinely assessed by the optical coherence tomography (OCT).

#### Health services utilisation

CCM aims at decreasing inappropriate use of the health care system by a proactive management. To assess if our intervention can fulfil this goal, we will assess patients' health service utilisation during the study period and the year before the study started. Patients will be asked to self-report all contacts to physicians in private practice, hospitals and emergency rooms. We will distinguish as far as possible between wet AMD associated consultations and consultations associated with other reasons/diseases.

The HMT enables patients to self perform a measurement of visual acuity. To assess whether this application increases the adherence, appointments triggered by the HMT will be assessed separately. The CCC will document for each contact the reason for encounter and the initiator of the encounter (patient/physician/chronic care coach/other).

#### Accordance to the CCM

##### Patients' perspective

Patients' assessment of the provided care will be assessed with the Patient Assessment of Chronic Illness Care (PACIC) which has been developed to assess congruency of provided health care to the CCM [24]. It is organized according to the key elements of the CCM and assesses the behavior of professionals and practice teams from a patient's perspective. The PACIC contains 20 items assessing 5 scale constructs: patient activation (assesses to what extent the patient was motivated and supported by the physician to initiate changes), delivery system design/decision support (assesses if the patient was supported e.g. by booklets and how satisfied he was with the organization of his care), goal setting/tailoring (assesses to what extent general instructions and suggestions were adapted to his personal situation), problem solving/contextual (assesses how the physician dealt with problems which interfered with achieving predefined goals), follow-up/coordination (addresses how frequently and consequently the whole process was followed-up). Recently, a German version of the PACIC has been validated in a sample of osteoarthritis patients [25]. The PACIC was also validated in a sample of diabetes patients [24] and its psychometric properties have been shown good in primary care patients with major depression [26].

##### Provider perspective

To assess accordance to the CCM of the health care provider's perspective, the Assessment of Chronic Illness Care (ACIC) [27] will be used. The ACIC is aimed at organizational teams to help identifying areas for improvement in their care for chronic illnesses and to evaluate the level and nature of improvements made in their system. It consists of 28 items covering the six areas of the CCM: Organization of the healthcare delivery system (6 items), community linkages (3 items), self-management support (4 items), decision support (4 items), delivery system design (6 items) and clinical information systems (5 items). Responses fall within four descriptive levels (D, C, B, A) of implementation ranging from D "little or none" to A, a "fully implemented" intervention. Within each of the four levels, respondents are asked to choose one of three ratings of the degree to which that description applies. The result is a 0-11 scale, with categories defined as follows: 0-2 (little or no support for chronic illness care), 3-5 (basic or intermediate support for chronic illness care), 6-8 (advanced support) and 9-11 (optimal, or comprehensive, integrated care for chronic illness). Subscale scores for the six areas are derived by summing the response. Bonomi et al. showed all six ACIC subscale scores to be responsive to health care quality-improvement efforts [27]. A translated and culturally adapted version into German has just been validated by the authors [28].

### Confounder control

Depression is highly prevalent among patients with chronic diseases and has been identified as important predictor for quality of life as well as clinical outcomes. It can also be an important confounder for the assessment of the success of interventions, aiming at improving quality of life. Therefore, we will assess depression by means of the Prime MD Patient Health Questionnaire (PHQ-9) [29]. The PHQ-9 is an established instrument to assess depression, especially in a primary care setting [7,30].

Furthermore, we assess the diabetes diagnosis as a potential confounder because of the diabetes patients' risk for diabetic retinopathy.

### Data collection

The CCCs will fill in the participants' names into a list in order of their inclusion and allocate a code to each patient. This code is also marked on all questionnaires and patient data which will be sent to the Institute of General Practice and Health Services Research at the University of Zurich. Therefore, the university only receives the patients' codes and has no access to their names.

Data regarding visual acuity assessed by the standardised ETDRS test and the clinical outcome OCT will be routinely assessed by the physicians. The NEI VFQ-25 will be provided to the patients by the trained CCC (assessed face-to-face, if this is not possible it will be assessed by telephone).

Patients will receive a questionnaire, containing sociodemographic variables, data about health service utilization, the PACIC [24] and the PHQ-9 [29], and a stamped envelope with the postal address of the university. The patients are asked to return this questionnaire in the envelope to the university. Health care providers will not get knowledge about the collected data to avoid any influence on the adherence, especially in the control group (via increased awareness by decreasing scores).

A second questionnaire is filled out by the physicians and/or the CCC for each participant regarding the actual ETDRS test values, central retinal thickness (via OCT), comorbidities, smoking status as a known risk factor [31,32] and medication. This questionnaire is marked as well with the patient's code and will be returned to the university in a stamped envelope. Additionally, the physicians fill out the ACIC.

Data regarding visual acuity, OCT and the NEI VFQ-25 will be assessed at baseline, after 6 and 12 months, the questionnaires at baseline and after 12 months. An independent research assistant of the university will enter the data directly into the SPSS program (version 18.0 or higher).

### Monitoring

At least two outreach visits will be performed in each centre, the first briefly after study onset and the second during the study year. The aim of these outreach visits are to assess if the ETDRS assessments will be conducted in a standardized way according the predefined specifications (adherence of correct distance of 4 m, use of standardized ETDRS charts etc.). Furthermore these visits aim to support the CCC and to reveal possible problems which might have occurred and to discuss and implement appropriate solutions. The outreach visits will be performed by a study coordinator.

### Sample size

The primary endpoint is the visual acuity. To asses visual acuity, we will perform a standardized ETDRS test. According to previous data, we assumed a mean of 48 and a standard deviation (SD) of 15 letters for the patients with AMD [33,34]. We hypothesized that a change of 5 letters can be achieved (of 70 letters in total, 5 per row, 14 rows), which can be regarded as clinically relevant. According to these assumptions, a number of 282 patients is needed to achieve a power of 80% and a significance level of 5%. Assuming a drop out rate of 25% the total sample size has to be 352. This number of patients would also be sufficient to detect a difference of 5 points in the NEI VFQ-25. According to the Manual of the National Eye Institute for the VFQ-25, version 2000 [21], Table eight (Sample sizes needed per group to detect differences in change over time between two experimental groups for the VFQ-25, repeated measures design), 161 patients in each treatment arm are needed to detect a minimum change in the VFQ-25 of 5 points, which has been defined as clinically relevant [35,36].

### Recruitment of centres

Around 20 specialised medical doctors (MDs) from 20 leading centres in Switzerland which provide therapy with anti-angiogenic drugs for AMD patients will be invited by a formal letter of the Institute of General Practice and Health Services Research of the University of Zurich to an information meeting. The content of the meeting is to provide the MDs with detailed information about the study, the specific intervention and the associated efforts for participation. If a centre agrees to participate in the study, it will be provided with all the required information and material and it will have to designate a CCC who will be trained in a one day session.

### Randomization

Eligible patients will be informed about the study by their physicians in the centres. After giving their informed consent, patients will be enrolled consecutively by the CCC in the study and allocated randomly in a 1:1 ratio to the intervention or control group (usual care, no intervention) (block randomization, stratified by study centre). A randomization list will be computed by means of STATA statistical software program version 11 and provided step by step for each patient to the centres/chronic care coach by telephone by the Institute of General Practice. We aim the physician to be blind regarding to the allocation.

### Statistical methods

To determine whether the intervention is associated with a change in the visual acuity score, we perform a 2-sided t-test to compare the mean ETDRS values in the intervention and the control group. Further analyses include the regression of ETDRS depending on a set of variables including treatment group, age, gender, disease duration and years of education, taking multiple observations over time into account.

The primary data analysis will follow the intention-to-treat (ITT) principle which means that the data will be analyzed according to the original treatment group assignment regardless of whether or not each individual actually received the assigned treatment. Corresponding to an intention-to-treat-analysis the data of drop out patients will be integrated into the follow up analyses. For missing values we will assume the mean of the value in the control group. A last observation carried forward approach (LOCF) is inappropriate, because the outcome would be overestimated as we investigate patients with a chronic degenerative ocular disease. We will also compare if there are any differences in the characteristics between the drop-out and the patients remaining in the study.

### Description of risks

Serious risks or undesired effects of the CCM or the assessment by questionnaires have not been described in the literature. There are no specific risks related to the study.

### Ethical principles

The study is being conducted in accordance with medical professional codex and the Helsinki Declaration as of 1996 as well as Data Security Laws and according to the Guidelines of Good Clinical Practice.

Study participation of patients is voluntary and can be cancelled at any time without provision of reasons and without negative consequences for their future medical care.

### Patient informed consent

Previous to study participation patients receive written and spoken information about the content and extent of the planned study; for instance about potential benefits for their health and potential risks. In case of acceptance they sign the informed consent form.

### Vote of the ethics committee

The study protocol has been approved by the ethics board of the Kanton Zurich on 17.12.2010 (KEK-ZH-NR: 2010-04391/1).

### Data security/disclosure of original documents

The patient names and all other confidential information fall under medical confidentiality rules and are treated according to the appropriate Federal Data Security Law.

All study related data and documents are stored on a protected central server of the University of Zurich. Only direct members of the internal study team can access the respective files.

Intermediate and final reports are stored in the office of the Institute of General Practice and Health Services Research at the Zurich University Hospital.

## Discussion

The CCM has achieved widespread acceptance as an evidence based template for the care of chronically ills. Wet AMD has the same characteristics as the diseases for which the CCM has been established as e.g. diabetes: Its natural course leads to a continuous loss in visual acuity. On the other hand there are established treatments available. The effectiveness of these treatments largely depends on a frequent application. Similar to diabetes, where a frequent follow-up and a proactive approach with action before the disease gets worse is effective, wet AMD has to be treated before visual acuity gets worse or completely lost. Therefore the characteristics of wet AMD qualify it as a "classic" chronic disease. Thus it can be assumed that some of the elements of the CCM, mainly the frequent follow up by a structured case management, will result in better clinical outcomes.

Multiple studies have shown that implementing CCM elements improve clinical outcomes as well as process parameters in different chronic diseases as osteoarthritis, depression or e.g. the cardiovascular risk profile of diabetes patients. This study will for the first time assess this approach in wet AMD. If our hypothesis will be confirmed, the implementation of this approach in routine care for patients with wet AMD should be considered.

## Competing interests

The authors declare that they have no competing interests.

## Authors' contributions

AF, KW, MW, TR designed the general idea and the study protocol, UH planned data analysis, TR secured funding for the trial, AF and KW drafted the manuscript. All authors read and approved the final manuscript.
